# Factors associated with the municipal provision of dental prostheses in the Brazilian Unified Health System

**DOI:** 10.1590/1807-3107bor-2026.vol40.074

**Published:** 2026-09-18

**Authors:** Tiago Schaffer RAMOS, Diógenes Dias OLIVEIRA, Caren Serra BAVARESCO, Flávio Renato Reis de MOURA

**Affiliations:** (a) Universidade Luterana do Brasil – Ulbra, Postgraduate Program in Dentistry, Canoas, RS, Brazil.; (b) Universidade Luterana do Brasil – Ulbra, Postgraduate Program in Dentistry, Canoas, RS, Brazil.; (c) Hospital Conceição, Porto Alegre, RS, Brazil.; (d) Universidade La Salle - Unilasalle, Postgraduate Program in Human Health and Development, Unilasalle, Canoas, RS, Brazil.

**Keywords:** Local Health Systems, Cities, Universal Healthcare, Oral Health, Dental Prosthesis

## Abstract

The aim of the present study was to investigate factors associated with the municipal provision of dental prostheses via the Brazilian Unified Health System (UHS). This cross-sectional study covered 5,565 Brazilian municipalities, utilizing data from a secondary database. The dependent variable was the municipal provision of dental prostheses. The independent variables were demographic and socioeconomic characteristics (regions of Brazil, number of residents in the municipality, health budget, gross domestic product [GDP] per capita, municipal human development index [MHDI], and Gini coefficient) and municipal structure of primary oral care (family health team [FHT] coverage, oral health team [OHT] coverage, OHT/FHT proportion) and secondary oral care (presence of dental specialties center [DSC], types of DSC, municipality employing at least one dentist specialized in dental prosthetics [DSDP], presence of regional dental prosthesis laboratory [RDPL]). Poisson regression with robust variance (p ≤ 0.05) was used for multivariate analysis. A total of 57% of Brazilian municipalities provided dental prostheses via the UHS. Municipalities in the Northeast region were 16% (PR = 1.16; 95%CI: 1.12-1.20) more likely to provide dental prosthetics via the UHS. Municipalities with a low GDP per capita (PR = 1.10; 95%CI: 1.08-1.11), the presence of a DSC (PR = 1.17; 95% CI: 1.15–1.19), the presence of a type III DSC [containing 7 or more dental offices] (PR = 1.06; 95%CI: 1.03–1.10), an employed DSDP (PR = 1.15; 95%CI: 1.12–1.18) and an RDPL (PR = 1.25; 95%CI: 1.23–1.27) were more likely to provide dental prostheses via the UHS. Demographic and socioeconomic factors and the available primary and secondary oral care structure influenced the municipal provision of dental prostheses via the UHS.

## Introduction

Edentulism is a serious oral health problem in the context of the global burden of disease.^1^ Tooth loss compromises the physiological functions of speech,^2^ mastication^3^ and digestion,^4^ as well as aesthetics, limiting social integration.^5^ The greater the number of missing teeth, the greater the negative impact on quality of life.^6^ This situation mainly affects individuals over 75 years of age, with an estimated worldwide prevalence of 22% (11% in North America, 16% in Asia-Oceania, 28% in Europe and 37% in South America).^9^ In Brazil, a nationwide oral health epidemiological survey conducted in 2010 showed that more than 90% of older adults needed dental prostheses and more than 60% already used complete upper dentures^10^. The 2023 Oral Health Survey demonstrated that, among older adults, 31% did not use upper dental prostheses and 52% did not use lower dental prostheses, despite a 36% prevalence of edentulism.^11^ Thus, tooth loss constitutes a significant social problem in Brazil^12-14^ and globally.^15-18^

As of 2023, Brazil had a population of 203 million^19^ and a gross domestic product (GDP) of R$9.9 trillion.^20^ The country had 43,286 Family Health Teams (FHT) (December 2020 data) and 35,827 Oral Health Teams (OHTs) (April 2024 data) providing primary care (www.egestorab.saude.gov.br),^21^ 1,210 Dental Specialties Centers (DSC) and 3,962 Regional Dental Prosthesis Laboratories (RDPL) providing secondary care, and 34 Centers for the Treatment of Lip and Palate Malformations (as of January 2025; https://datasus.saude.gov.br/cnes-estabelecimentos^22^) at the tertiary level, all integrated to the Brazilian Unified Health System (UHS).

The UHS (in Portuguese, *Sistema Único de Saúde*, SUS) is governed by three inter-federative entities, represented at the federal level by the Ministry of Health (MoH) and at the state and municipal levels by the respective departments of health. Its core principles are to provide health care to the Brazilian population in a universal, comprehensive, equitable manner, and its core functions are health promotion, disease prevention, and the recovery of health.^23^ Within this structure, the provision of dental prostheses via the UHS must be organized and financed by the inter-federative entities under the leadership of the MoH. In 2004, the Ministry implemented the Brazilian National Oral Health Policy (BNOHP), with the aim of consolidating and expanding the provision of dental prostheses at primary care centers (*Unidades Básicas de Saúde*)^24^ and DSCs.

The MoH regulates funding for dental prostheses via the UHS through ministerial ordinances. Ordinance 1,572 of 2004 established a value of US$5.30 for complete mandibular and maxillary prostheses. RDPLs were implemented in 2006 by Ordinance 599, which also established forms of funding for dental prostheses. In 2009, Ordinance 2,734 established a value of US$10.50 for complete mandibular and maxillary prostheses, removable partial mandibular and maxillary prostheses, and crown/intraradicular and adhesive/fixed prostheses (per element). Ordinance 1,825 was issued in 2012, raising the value to US$26.30. Technical Note No. 20, published in 2021,^25^ again updated the level of funding provided to RDPLs by production range: US$1,316 for those fabricating 20 to 50 prostheses per month, US$2,105 from 51 to 80 prostheses per month, US$3,158 from 81 to 120 prostheses per month, and US$3,947 for those with an output above 120 prostheses/month. In 2023, the MoH readjusted and unified the level of funding for all types of dental prostheses provided by the UHS at US$39.50.^26^

Despite investments for the provision of dental prostheses via the UHS, Northern Brazil is the region of the country with the lowest growth trend,^27^ the greatest need and the lowest delivery rate.^28^ The supply of dental prostheses in primary care is unevenly allocated across Brazil, with fewer than 50% of primary health centers that have an OHT providing these devices.^29^ The allocation and availability of RDPLs is also uneven, with a greater concentration in the Northeast region, suggesting that several aspects may be related to the supply of dental prostheses,^28^ such as the population size of each municipality, setting (urban or rural), and the municipal budget allocated to health care (availability of healthcare providers and health facilities that offer dental prosthetics). In addition, although greater OHT and DSC coverage at the municipal level improves the supply of dentures (complete dental prostheses), it may exacerbate racial inequalities if administrators fail to prioritize oral health care for specific groups, such as the Black population.^30^

Studies have explored the process of providing dental prostheses via the UHS^30,31^ with the aim of assisting managers, workers and the population in implementing this service. Furthermore, factors such as the municipal government decentralizing the supply of dental prostheses via primary care,^24^ availability of qualified human resources,^32^ better socioeconomic and educational status of the population,^33^ as well as the quality of service management and organization,^32^ may all contribute to access to dental prosthetics services. However, there is a lack of studies in the literature using census data to investigate municipal-level factors that can contribute to the provision of dental prostheses via the UHS. Unknown factors may influence policies related to oral rehabilitation in the UHS.^28^ Therefore, the aim of the present study was to investigate factors associated with the municipal provision of dental prostheses via the UHS. The null hypothesis is that demographic and socioeconomic factors and the structure of primary and secondary oral care would not influence the provision of dental prostheses in Brazilian municipalities.

## Methods

### Design and sample

A cross-sectional study was conducted following the Strengthening the Reporting of Observational Studies in Epidemiology (STROBE) guidelines. A census of all 5,570 Brazilian municipalities was conducted; five were ultimately excluded due to incomplete data on Municipal Human Development Index (MHDI).

### Variables and data collection

Data were collected in February and March 2024. The outcome variable was municipalities that provided dental prostheses via the UHS in the year 2023 (Figure). Municipalities that provided at least 12 dental prostheses throughout the year 2023 (i.e., at least one per month) constituted the outcome variable. The types of dental prostheses offered by the UHS were determined according to MoH Ordinance No. 6/2017 (Table 1).

**Figure:**
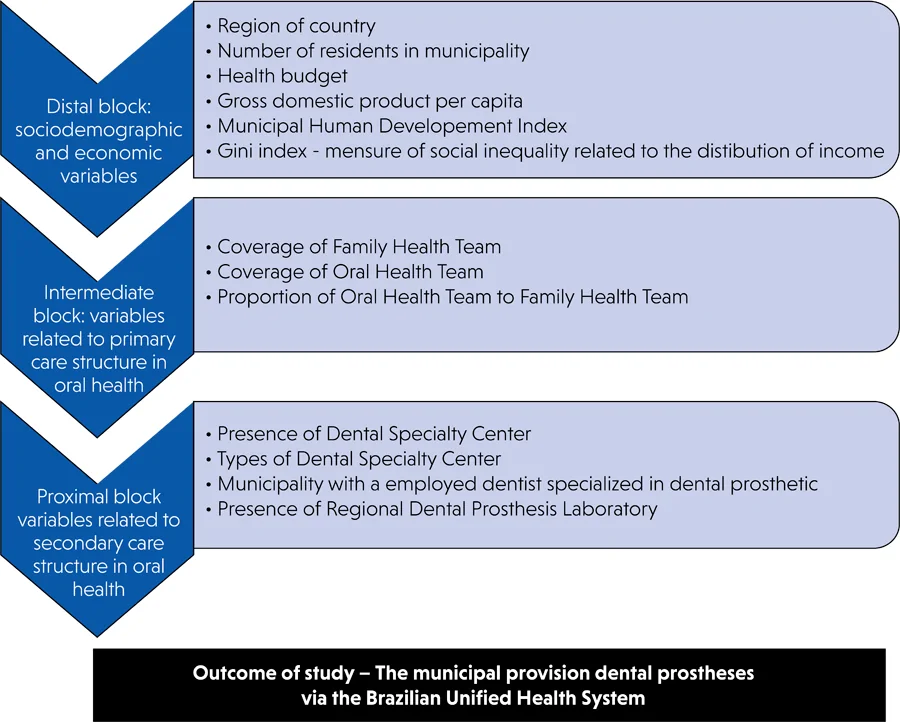
Hierarchical theoretical model for multivariate analysis.

**Table 1:** Types of dental prosthesis established by Ministerial Ordinance Nº 6 of 2017.

Procedure/Type of Prosthesis	SIGTAP-OMP/SUS* Code
Complete Mandibular Prosthesis	07.01.07.012-9
Complete maxillary prosthesis	07.01.07.013-7
Removable partial mandibular prosthesis	07.01.07.009-9
Removable partial maxillary prosthesis	07.01.07.010-2
Fixed/adhesive crown/Intra-radicular prostheses (per element)	07.01.07.014-5

Brazil, 2017; SIGTAP-OMP/SUS: Management system for the table of procedures, medications, orthoses, prostheses, and special materials of the Universal Healthcare System.

The independent variables (Figure) were divided into three blocks: demographic and socioeconomic variables, primary oral care structure, and secondary oral care structure. The demographic and socioeconomic variables were: regions of Brazil (North, Northeast, Central-West, Southeast and South), number of residents in the municipality, MHDI (an indicator of human development considering dimensions of education, income and longevity), Gross Domestic Product (GDP) per capita (an indicator that assesses economic performance), Gini coefficient (an indicator that measures the income concentration of a particular group, ranging from 0 to 1, with values closer to zero indicating income equality and those closer to one indicating inequality) and municipal health budget. The primary oral care structure variables were FHT coverage, OHT coverage within FHTs, and OHT/FHT ratio. Finally, the secondary oral care structure group comprised presence of DSC, type of DSC (Type I, 3 dental offices; Type II, 4 to 6 dental offices; Type III, 7 or more dental offices), the presence of a dentist specialized in dental prosthetics (DSDP) employed by the municipality (according to the Brazilian Classification of Dental Surgeon Occupations, code 2232-56) and the presence of an RDPL.

The dependent and independent variables were collected from public databases managed by the Brazilian government, as follows: number of dental prostheses produced, regions of Brazil, population size of municipality, GDP per capita, Gini coefficient, presence and type of DSC, DSDP employed by municipality, and presence of RDPL were collected from the UHS Information Technology Department platform (DATASUS, www.datasus.gov.br), using the public-domain TABNET application that allows data to be exported in Microsoft Excel format. The variables MHDI and municipal health budget were collected from the United Nations Development Program (UNDP, www.undp.org.br/pt/brazil) and the Brazilian federal government’s Public Health Budget Information System (SIOPS, https://www.gov.br/saude/pt-br/acesso-a-informacao/siops), respectively. Finally, the variables FHT coverage and OHT coverage were collected from the Primary Care Information and Management platform (e-gestorab, https://egestorab.saude.gov.br). All data were collected by a single researcher and verified during creation of the database.

### Statistical analysis

A database was created in Microsoft Excel consisting of the dependent outcome variable and independent variables (covariates). Data were combined using the numerical codes of the municipalities to construct the database, which was then exported to the Statistical Package for the Social Sciences (SPSS) version 20.0.

Prior to statistical analysis, the outcome variable was categorized dichotomously as a) municipalities that do not offer dental prostheses via the UHS and b) those that do. The covariates were categorized into regions of Brazil: a) North, b) Northeast, c) Southeast, d) South and e) Central-West. Municipal population (median, 10,934), municipal health budget (median, US$10,347,331), GDP per capita (median, US$2,214.75), MHDI (median, 0.665), and Gini coefficient (median, 0.502) were categorized by the median. Presence of a DSC and presence of an RDPL within municipal limits were categorized as a) absent or b) present. DSCs were categorized as 1) Type I, 2) Type II or 3) Type III. The covariates FHT coverage and OHT coverage were categorized as ≤ 80% and > 80%,^34^ and the OHT/FHT ratio was categorized as a) <1:1 or b) ≥1:1. The covariate “municipality employs a DSDP” was categorized as a) no or b) yes.

Descriptive analyses were performed, with the calculation of absolute (n) and relative (%) frequencies. The chi-square test was used for bivariate analyses to determine associations between the covariates and outcome variable (p < 0.05). A hierarchical model composed of levels was developed for the multivariate analysis (Figure) using the backward stepwise approach. The distal level was composed of the block of demographic and socioeconomic variables, the intermediate level was composed of the block of primary oral health care structure variables, and the proximal level was composed of the block of secondary oral health care structure variables^35^. Poisson regression with robust variance^36^ was used to calculate the effect measure: prevalence ratio (PR) with confidence intervals (CIs) (p < 0.05).

As the study involved the collection of publicly available data of a secondary nature, in accordance with the ethical precepts of Resolution 466/2012 of the Brazilian National Health Council^37^ and the Declaration of Helsinki,^38^ no approval from a Research Ethics Committee was required.

## Results

### Descriptive and bivariate analyses

Among the 5,570 municipalities of Brazil, 5,565 had MHDI data available and were thus included in the study. Of these, 3,168 (56.9%) provided dental prostheses via the UHS (Table 2) and all had provided more than 12 prostheses in the year 2023. A simple majority of municipalities were located in the Northeast region (n = 1,794; 32.2%). As also shown in Table 2, 50% of the municipalities had a population of >10,935 (n = 2,782; 50.0%), a health budget > US$10,347,331 (n = 2,781; 50.0%), GDP per capita > US$2,214 (n = 2,781; 50.0%), MHDI ≤ 0.665 (n = 2,790; 50.1%), and Gini coefficient > 0.502 (n = 2,797; 50.3%).

**Table 2:** Descriptive and bivariate analyses of demographic and socioeconomic variables (distal level), variables related to structure of primary care in oral health (intermediate level), and variables related to structure of secondary care in oral health (proximal level).

Variables	n	%	Municipalities that offer dental prostheses		
			n	(%)	p-value
	5565	100	3168	56,9	
Distal level: demographic and socioeconomic variables					
Regions of country					
North	449	8.1	216	48.1	0.00
Northeast	1794	32.2	1294	72.1	
Southeast	1668	30	922	55.3	
South	1188	21.3	454	38.2	
Central West	466	8.4	282	60.5	
Number of residents in municipality					
≥ 10,934	2783	50	1485	53.4	0.00
≤ 10,935	2782	50	1683	60.5	
Health budget*					
≥ 10,347,331	2784	50	1495	53.7	0.00
> 10,347,331	2781	50	1673	60.2	
GDP per capita^1^					
≥ 2,214.75	2784	50	1777	63.8	0.00
> 2,214.75	2781	50	1391	50	
MHDI^2^					
≥ 0.665	2790	50.1	1773	63.5	0.00
> 0.665	2775	49.9	1395	50.3	
GINI^3^					
> 0.502	2797	50.3	1709	61.1	0.00
≥ 0.502	2768	49.7	1459	52.7	
Intermediate level: variables related to structure of primary care in oral health					
Coverage of FHT					
≥ 80%	941	16.9	454	48.2	0.00
> 80%	4624	83.1	2714	58.7	
Coverage of OHT					
≥ 80%	1541	27.7	887	57.6	0.56
> 80%	4024	72.3	2281	56.7	
Proportion of OHT to FHT					
Without OHT	237	4.3			
01:02	2487	44.7	1350	54.3	0.00
01:01	2841	51.1	1761	62	
Proximal level: Variables related to structure of secondary care in oral health					
Presence of DSC					
No	4576	82.2	2358	54.1	0.00
Yes	989	17.8	753	78	
Types of DSC					
Type I	466	47.1	365	78.3	0.00
Type II	403	40.7	294	73	
Type III	120	12.1	108	90	
Municipality with employed DSDP					
No	5107	91.8	464	71.3	0.00
Yes	458	8.2	303	89.6	
Presence of RDPL					
No	3524	63.3	125	52.1	0.00
Yes	2041	36.7	642	85.7	

Brazil, 2023; Health budget = U$1,00 = R$5,7; GDP: Gross domestic product; MHDI: Municipal Human development index; GINI - Measure of social inequality related to the distribution of income; FHT - Family Health Team; OHT - Oral Health Team; DSC - Dental Specialty Center; DSDP:Dentist Specialized in Dental Prosthetics; RDPL: Regional Dental Prosthesis Laboratory..

Most municipalities had > 80% primary health team coverage (FHT: n = 4,624, 83.1%; OHT: n = 4,024, 72.3%). A 1:1 OHT/FHT ratio was found in 51.1% of municipalities (n = 2,841). Overall, 82.2% (n = 4,576) of Brazilian municipalities did not have a DSC; among those that did, the majority were Type I centers (n = 466; 47.1%). More than 90.0% (n = 5,107) of municipalities did not have a prosthodontist employed by the UHS, and 63.0% (n = 3,524) did not have an RDPL operating within municipal limits (Table 2).

The results of bivariate analysis (Table 2) revealed significant associations (p < 0.05) between the outcome variable (provision of dental prostheses via the UHS in 2023) and the demographic and socioeconomic variables. For instance, the Northeast region had the highest number of municipalities that provided dental prostheses via the UHS (n = 1,294; 72.1%). Municipalities with a larger number of residents (n = 1,683; 60.5%) and larger health budget (n = 1,673; 60.2%) provided more dental prostheses than those with a smaller population and smaller budget. The municipalities with the lowest GDP (n = 1,777; 63.8%) were more likely to provide dental prostheses than those with the highest GDP (n = 1,391; 50.0%). Regarding variables related to the municipal primary health care structure, the outcome variable was significantly associated (p < 0.05) with larger population covered by FHTs (n = 2,714; 58.7%) and having a 1:1 OHT/FHT ratio (n = 1,761; 62.0%). Population coverage by OHTs was not associated with the outcome variable (p > 0.05). Regarding variables related to the municipal secondary health care structure, presence of any DSC, presence of a Type I DSC, having a prosthodontist employed via the UHS, and having an RDPL within municipal limits were all significantly associated (p < 0.05) with the provision of dental prostheses via the UHS.

### Multivariate analysis

The multivariate analysis (Table 3) revealed that municipalities located in the Northeast region were 16% more likely (PR = 1.16; 95%CI: 1.12–1.20; p = 0.00) to provide dental prostheses via the UHS compared to those in the North region. Furthermore, municipalities with a population of > 10,935 were 2% more likely (PR = 1.02; 95%CI: 1.00–1.04; p = 0.01) to provide dental prostheses. The health budget variable lost its strength of association and did not demonstrate a significant size effect (p = 0.19). Municipalities with lower GDP per capita were 10% more likely (PR = 1.10; 95%CI: 1.08–1.11; p = 0.00) to provide dental prostheses via the UHS. Municipalities with a lower MHDI (PR = 1.05; 95%CI: 1.02–1.07; p = 0.00) and higher Gini coefficient (PR = 1.03; 95%CI: 1.01–1.05; p = 0.02) were more likely to provide dental prostheses via the UHS compared to their reference categories.

**Table 3:** Unadjusted and adjusted analyses the municipal provision of dental prostheses in Brazilian Unified Health System according to distal, intermediate, and proximal levels.

Variables	Unadjusted		Adjusted	
	PR (95%CI)	p-value	PR (95%CI)	p-value
Distal level: demographic and socioeconomic variables				
Regions of country				
North	1		1	
Northeast	1.16 (1.12–1.20)	0.00	1.16 (1.12–1.20)	0.00
Southeast	1.05 (1.01–1.09)	0.01	1.05 (1.01–1.09)	0.00
South	0.93 (0.90–0.97)	0.00	0.94 (0.90–0.97)	0.00
Central West	1.08 (1.04–1.13)	0.00	1.09 (1.04–1.13)	0.00
Number of residents in municipality				
≥ 10,934	1		1	
≤ 10,935	1.05 (1.03–1.06)	0.00	1.02 (1.00–1.04)	0.01
Health budget				
≥ 10,347,331	1		1	
> 10,347,331	1.04 (1.02–1.06)	0.00	1.02 (0.99–1.04)	0.19
GDP per capita				
≥ 2 ,214.75	1.09 (1.07–1.11)	0.00	1.10 (1.08–1.11)	0.00
> 2,214.75	1		1	
Municipal HDI^2^				
≥ 0.665	1.09 (1.07–1.11)	0.00	1.05 ( 1.2–1.07)	0.00
> 0.665	1		1	
GINI				
> 0.502	1.05 (1.04–1.07)	0.00	1.03 (1.01–1. 05)	0.02
≥ 0.502	1		1	
Intermediate level: Variables related to structure of primary care in oral health				
Coverage of FHTs				
≥ 80%	1		1	
> 80%	1.07 (1.05–1.10)	0.00	1.07 (1.04–1.09)	0.00
Coverage of OHTs				
≥ 80%	1		1	
> 80%	0.99 (0.98–1.01)	0.55	0.99 (0.97–1.01)	0.49
Proportion of OHTs to FHTs				
01:02	1		1	
01:01	1.05 (1.03–1.07)	0.00	1.05 (1.03 -1. 07)	0.00
Proximal level: Variables related to structure of secondary care in oral health				
Presence of DSC				
No	1		1	
Yes	1.15 (1.13–1.18)	0.00	1.17 (1.15–1.19)	0.00
Types of DSC				
Type I	1		1	
Type II	0.97 (0.94–1.00)	0.07	0.97 (0.94–1.00)	0.06
Type III	1.06 (1.03–1.10)	0.00	1.06 (1.03–1.10)	0.00
Municipality with employed DSDP				
No	1		1	
Yes	1.22 (1.20–1.25)	0.00	1.15 (1.12–1.18)	0.00
Presence of RDPL				
No	1		1	
Yes	1.27 (1.25–1.29)	0.00	1.25 (1.23–1.27)	0.00

Brazil, 2023; PR: prevalence ratio; CI: confidence interval; GDP: Gross domestic product; MHDI: Human development index; GINI: Measure of social inequality related to the distribution of income; FHT: Family Health Team; OHT: Oral Health Team; DSC: Dental Specialty Center; DSDP: Dentist Specialized in Dental Prosthetics; RDPL: Regional Dental Prosthesis Laboratory.

Table 3 also shows that municipalities with FHT coverage above 80% were 7% more likely (PR = 1.07; 95%CI: 1.04–1.09; p = 0.00) and municipalities with a 1:1 OHT/FHT ratio were 5% more likely (PR = 1.05; 95%CI: 1.03–1.07; p = 0.00) to provide prostheses via the UHS compared to municipalities with coverage below 80% and a 1:2 OHT/FHT ratio, respectively. The presence of a DSC in the municipality increased the likelihood of the outcome by 17% (PR = 1.17; 95 CI: 1.15–1.19; p = 0.00). Furthermore, municipalities that hosted Type III DSCs, those with a DSDP employed by the UHS, and those with an RDPL were 6% (PR = 1.06; 95%CI: 1.03–1.10; p = 0.00), 15% (PR = 1.15; 95%CI: 1.12–1.18; p = 0.00), and 25% (PR = 1.25; 95%CI: 1.23–1.27; p = 0.00) more likely to provide dental prostheses via the UHS, respectively, compared to their reference categories (municipalities with a Type I DSC, those without a DSDP employed by the UHS, and those without an RDPL).

## Discussion

Nationwide oral health surveys conducted in Brazil in 2003 and 2010 demonstrated the need for rehabilitation of the population affected by tooth loss^10,39^. In response to this situational diagnosis, in 2004 the MoH instituted the BNOHP, which established the provision of dental prostheses via the UHS. In 2009 and 2023, the Ministry further published ordinances which address the form of funding allocated by the Brazilian federal government for this purpose.

Twenty years after the BNOHP entered into force, our study found that 3,168 (56.9%) of the country’s municipalities provided dental prostheses via the UHS. We found that municipalities located in the Northeast region, as well as those with a larger population (≥ 10,935), lower GDP per capita (≤ 2,214.75), lower MHDI (≤ 0.665) and higher Gini coefficient (> 0.502), were more likely to provide dental prostheses. These results show that municipalities with greater socioeconomic limitations have a greater likelihood of providing dental prostheses free at the point of care to their populations, which is consistent with the core UHS principle of equity. In a previous study that used variables similar to those of the present investigation, municipalities with a lower MHDI, lower income per capita and higher mean number of OHTs per 10,000 residents performed better in terms of providing specialized dental services (DSCs).^40^

Moreover, structural factors at the municipal level, such as greater population coverage by FHTs (> 80%), having a 1:1 OHT/FHT ratio, having any DSC or a Type III DSC, employing a specialist prosthodontist and having implemented an RDPL with federal funding were associated with greater likelihood of providing dental prostheses via the UHS. Thus, our study corroborates the findings of previous investigations^32^ that a well-structured oral health network in primary care, with its workflow incorporated into the family health strategy, a dedicated oral health team for each primary care unit (1:1 OHT:FHT ratio) and integration with the broader healthcare network (dental specialties center operating at the secondary care level)^40^may be essential factors for the expansion of provision of dental prostheses via the UHS. Although this provision may be reducing social inequities, such inequities may still persist for specific groups, such as the Black population.^30^ Our findings are essential to inform a customized analysis by administrators at the federal, state and municipal levels, as they reveal fundamental factors for the planning and implementation of dental prosthetic services via the UHS.

Our results also demonstrate that the BNOHP is benefiting socioeconomically disadvantaged municipalities when we analyze factors such as region, GDP per capita, municipal MHDI and Gini coefficient. These factors demonstrate that it is possible for municipal administrators, supported by state and federal administrators, to conceive, plan and implement the provision of dental prostheses via the UHS in socioeconomically vulnerable municipalities which are struggling to reduce social inequalities among Brazilians, especially for residents of the Northeast region, where the self-perceived need for dental prosthetics was > 60% in the 2023 Oral Health Survey.^11^ Thus, it is essential for administrators to have political will and knowledge of the public policies proposed by the federal government. Moreover, monitoring and follow-up by social control entities (health councils) is required at the municipal, state and national levels to ensure implementation of the BNOHP. Providing dental prosthetics services to the population is possible by consolidating and improving the oral health network, structuring its secondary level of care through implementation of DSCs. In the 20th anniversary year of the BNOHP, our results also indicate that public resources allocated by inter-federative entities are being used to reverse the poor oral health situation of socially disadvantaged communities.

A study conducted in Brazil based on data from the Program for the Improvement and Access to Quality Primary Care^32^ showed that the provision of dental prostheses in primary care is also feasible. The same study found that OHTs which employ dental practitioners admitted through public service examinations and practitioners involved in continuing education were more likely to provide dental prostheses. Another study^41^ using data from the second cycle of the Brazilian Program for the Improvement and Quality of Dental Specialty Centers found that 24% of individuals underwent prosthetic rehabilitation at such centers. Factors such low educational attainment, residing in a municipality that has a DSC and receiving care at a DSC located in an inland municipality rather than a major urban center contributed to the study participants achieving their desired prosthetic rehabilitation via the UHS.

A previous study conducted by our working group demonstrated that municipalities hosting Type III DSCs, which employ specialists on the municipal payroll, are more likely to provide oral health services via the UHS^35^. A recent study demonstrated that municipalities with DSCs and more than 40 OHTs improve access to dental prosthetics in the UHS; however, social inequalities persist^30^. The present study found that presence of a Type III DSC and presence of a specialist prosthodontist on staff improved adherence to the MoH funding structure, enabling implementation of an RDPL and increasing the likelihood of the municipality offering a dental prosthetics service. Such findings demonstrate that structural factors and municipal adherence to the BNOHP funding scheme are essential to the provision of dental prosthesis via the UHS, which is consistent with our research hypothesis.

Structuring public policies for oral health and promoting oral health by providing dental prostheses are of extreme importance to meeting the demand found in the 2023 Oral Health Survey, which reported that 23% of older adults in Brazil needed dental prostheses, with this figure reaching 30% among residents of inland municipalities of the Northeast region^11^. We suggest that our findings and those of other studies^31,32^ can contribute to the design, implementation and monitoring of dental prosthetics services via the UHS. From a global standpoint, governments and communities in other countries that have health policies, socioeconomic conditions and oral health scenarios similar to those of Brazil may also use the factors discussed in our study to implement oral health policies or improve the quality of existing ones.

Our work shows that larger municipal health budgets and OHT coverage above 80% were not associated with the outcome of interest. It is possible that municipal managers are prioritizing hospital-based care and emergency services and strengthening the demands of primary care in the general and oral health fields with basic procedures (e.g., dietary/oral hygiene counseling, supragingival periodontal treatment, restorative treatment, tooth extraction, etc.), which may explain our results demonstrating a large number of municipalities (72%) with OHT coverage (> 80%). Furthermore, fewer than 50% of Brazilian municipalities provide dentures at primary care facilities.^29^

The strengths of this study include the use of national databases to conduct the census and the use of Poisson regression with robust variance in the multivariate analysis, determining the prevalence ratio of the outcome and enabling correction of confidence intervals that could be wider. Moreover, this is the first study conducted in Brazil to assess municipal-level factors associated with provision of dental prostheses via the UHS. As limitations, our study did not assess the number and types of prostheses (total, partial, etc.) delivered in 2023 or whether the population needs for dental prostheses reported in the 2023 Oral Health Survey were effectively met. Moreover, the cross-sectional design impedes causal inferences. We also used secondary data that may have been affected by under- or overreporting. Although the study indicated that municipalities with unfavorable economic factors offer prosthetic services through their public healthcare facilities, this does not ensure that the vulnerable population is accessing such services or that social inequalities are being reduced.^41^ Despite these limitations, our study has a national scope and methodological design that sought to avoid information bias. Furthermore, it contributes greatly to the literature and consolidates important data for administrators, workers and instances of social control (such as health councils) regarding the provision of dental prosthetic rehabilitation services to the Brazilian population. Although the present study does not exhaust this subject, it may assist in the design of future research addressing factors associated with the longevity of prostheses obtained through the UHS.

Lastly, there is much to be done specifically with regards to combating edentulism as a public health problem. A recent study demonstrated that countries with better social macro determinants (governance, macroeconomic policy, public policies, social policies and social/cultural values) had higher rates of prevalence, incidence and years lived with disability due to edentulism.^1^. Therefore, oral health is closely related to the global burden of disease and should be a priority in the discussion of public policies.

## Conclusion

Demographic and socioeconomic factors (being located in the Northeast region, large municipal population, low GDP per capita, low MHDI, greater Gini coefficient) and factors related to primary and secondary care structure (greater FHT coverage, equal OHT/FHT ratio, having a DSC, having a Type III DSC, employing a specialist prosthodontist, presence of an RDPL) were all associated with provision of dental prostheses via the UHS. Conversely, the covariates larger municipal health budget and greater OHT coverage were not associated with this outcome. These findings should help inform administrators, healthcare workers and those involved in social control of the system, assisting in the implementation of dental prosthetic rehabilitation services and contributing to the quality of life of users of the Brazilian Unified Health System.

## Data Availability

The datasets generated during and/or analyzed during the current study are available from the corresponding author on reasonable request.

## References

[1] Fagundes ML, Júnior OL, Hugo FN, Kassebaum NJ, Giordani JM (2024). Distribution of Edentulism by the macro determinants of health in 204 countries and territories: an analysis of the global burden of disease study. J Dent.

[2] Hugo FN, Hilgert JB, Sousa ML, Silva DD, Pucca GA (2007). Correlates of partial tooth loss and edentulism in the Brazilian elderly. Community Dent Oral Epidemiol.

[3] Techapiroontong S, Limpuangthip N, Tumrasvin W, Sirotamarat J (2022). The impact of poor dental status and removable dental prosthesis quality on body composition, masticatory performance and oral health-related quality of life: a cross-sectional study in older adults. BMC Oral Health.

[4] Dibello V, Zupo R, Sardone R, Lozupone M, Castellana F, Dibello A (2021). Oral frailty and its determinants in older age: a systematic review. Lancet Healthy Longev.

[5] Nordenram G, Davidson T, Gynther G, Helgesson G, Hultin M, Jemt T (2013). Qualitative studies of patients' perceptions of loss of teeth, the edentulous state and prosthetic rehabilitation: a systematic review with meta-synthesis. Acta Odontol Scand.

[6] Gerritsen AE, Allen PF, Witter DJ, Bronkhorst EM, Creugers NH (2010). Tooth loss and oral health-related quality of life: a systematic review and meta-analysis. Health Qual Life Outcomes.

[7] Schierz O, Baba K, Fueki K (2021). Functional oral health-related quality of life impact: A systematic review in populations with tooth loss. J Oral Rehabil.

[8] Emami E, Souza RF, Kabawat M, Feine JS (2013). The impact of edentulism on oral and general health. Int J Dent.

[9] Borg-Bartolo R, Roccuzzo A, Molinero-Mourelle P, Schimmel M, Gambetta-Tessini K, Chaurasia A (2022). Global prevalence of edentulism and dental caries in middle-aged and elderly persons: a systematic review and meta-analysis. J Dent.

[10] Pesquisa Nacional de Saúde Bucal (2012). SB Brasil 2010: resultados principais.

[11] Pesquisa Nacional de Saúde Bucal (2025). SB Brasil 2023: relatório final.

[12] Peres MA, Barbato PR, Reis SC, Freitas CH, Antunes JL (2013). Tooth loss in Brazil: analysis of the 2010 Brazilian Oral Health Survey. Rev Saude Publica.

[13] Azevedo MS, Correa MB, Azevedo JS, Demarco FF (2015). Dental prosthesis use and/or need impacting the oral health-related quality of life in Brazilian adults and elders: results from a National Survey. J Dent.

[14] Cortez GF, Barbosa GZ, Tôrres LH, Unfer B (2023). Reasons for and consequences of tooth loss in adults and elderly people in Brazil: a qualitative metasynthesis. Cien Saude Colet.

[15] Kassebaum NJ, Bernabé E, Dahiya M, Bhandari B, Murray CJ, Marcenes W (2014). Global Burden of severe tooth loss: a systematic review and meta-analysis. J Dent Res.

[16] Zhang Y, Leveille SG, Shi L (2022). Multiple chronic diseases associated with tooth loss among the US adult population. Front Big Data.

[17] Kassebaum NJ, Smith AG, Bernabé E, Fleming TD, Reynolds AE, Vos T (2017). Global, regional, and national prevalence, incidence, and disability-adjusted life years for oral conditions for 195 countries, 1990-2015: a systematic analysis for the global Burden of diseases, injuries, and risk factors. J Dent Res.

[18] Peltzer K, Hewlett S, Yawson AE, Moynihan P, Preet R, Wu F (2014). Prevalence of loss of all teeth (edentulism) and associated factors in older adults in China, Ghana, India, Mexico, Russia and South Africa. Int J Environ Res Public Health.

[19] Instituto Brasileiro de Geografia e Estatística (2023). População.

[20] International Monetary Fund (2023). World economic outlook: navagation global divergences.

[21] Ministério da Saúde (BR), Secretaria de Atenção Primária à Saúde (2020). Painéis de indicadores de saúde bucal.

[22] Ministério da Saúde (BR), Departamento de Informática do Sistema Único de Saúde (2020). TabNet.

[23] Senado Federal (BR) (1988). Constituição da República Federativa do Brasil - 1988. Diario Oficial Uniao.

[24] Silva MF, Darabansk JA, Núgoli VZ, Simões AC, Cavalcante DFB, Baldani MH (2023). Oferta de prótese dentária na atenção básica: expansão e diferenças regionais no Brasil. Rev Contexto Saude.

[25] Ministério da Saúde (BR), Secretaria de Atenção Primária à Saúde (2023). Coordenação Geral de Saúde Bucal Nota técnica N o 20/2021- CGSB/DESF/SAPS/MS.

[26] Ministério da Saúde (BR) Portaria Ministerial GM/MS N o 1.924, de 17 de novembro de 2023. Altera a Portaria de Consolidação GM/MS nº 6, de 28 de setembro de 2017, para reajustar os valores dos incentivos financeiros das Equipes de Saúde Bucal - eSB, das Unidades Odontológicas Móveis - UOM, dos Laboratórios Regionais de Próteses Dentárias - LRPD e dos Centros de Especialidades Odontológicas - CEO segundo os critérios estabelecidos pela Politica Nacional de Atenção Básica e pela Politica Nacional de Saúde Bucal.

[27] Vieira MF, Marques PS, Figueiredo DR, Carcereri DL, Cascaes AM (2023). Production of dental prosthetics in the SUS in Brazilian older population and impact of the covid-19 pandemic. Rev Saude Publica.

[28] Aguiar VR, Celeste RK (2015). The need for, and allocation of, regional prosthodontics laboratories in Brazil: an exploratory study. Cien Saude Colet.

[29] Cunha MA, Matta-Machado AT, Lucas SD, Abreu MH (2018). Availability of dental prosthesis procedures in Brazilian Primary Health Care. BioMed Res Int.

[30] Bomfim RA, Lucena EH, Cavalcanti YW, Celeste RK (2023). Racial inequality in complete dental prosthesis delivered: can public services reduce inequities?. Clin Oral Investig.

[31] Cunha IP, Lacerda VR, Silva MF, Bomfim RA (2023). Associated factors of prosthetic rehabilitation in specialized dental care in Brazil: a cross-sectional study. BMC Res Notes.

[32] Cunha MA, Vettore MV, Santos TR, Matta-machado AT, Lucas SD, Abreu MHNG (2020). The role of organizational factors and human resources in the provision of dental prosthesis in primary dental care in Brazil. Int J Environ Res Public Health.

[33] Giordani JM, Slavutzky SM, Koltermann AP, Pattussi MP (2011). Inequalities in prosthetic rehabilitation among elderly people: the importance of context. Community Dent Oral Epidemiol.

[34] Stein C, Santos KW, Condessa AM, Celeste RK, Hilgert JB, Hugo FN (2019). Presence of Specialized Dentistry Centers and the relationship with dental extractions in the oral healthcare network in Brazil. Cad Saude Publica.

[35] Oliveira DD, Vargas IA, Busato AL, Brondani M, Bavaresco CS, Moura FR (2022). Factors associated with the municipal provision of orthodontics in the Brazilian Unified Health System. Community Dent Health.

[36] Coutinho LM, Scazufca M, Menezes PR (2008). Methods for estimating prevalence ratios in cross-sectional studies. Rev Saude Publica.

[37] Ministério da Saúde (BR) (2012). Resolução n o 466, de 12 de dezembro de 2012. Aprovar as seguintes diretrizes e normas regulamentadoras de pesquisas envolvendo seres humanos.

[38] Word Medical Association (2013). Word Medical Association Declaration of Helsinki. Ethical principles for medical research involving human subjects. JAMA.

[39] Ministério da Saúde (BR) (2004). Pesquisa Nacional de Saúde Bucal - SB Brasil 2003: condições de saúde bucal da população brasileira 2002-2003: resultados principais.

[40] Galvão MH, Roncalli AG (2021). Performance of Brazilian municipalities in the supply of specialized oral health services. Cad Saude Publica.

[41] Freire DE, Freire AR, Lucena EH, Cavalcanti YW (2021). Acesso em saúde bucal no Brasil: análise das iniquidades e não acesso na perspectiva do usuário, segundo o Programa de Melhoria do Acesso e da Qualidade da Atenção Básica, 2014 e 2018. Epidemiol Serv Saude.

